# Multidisciplinary Management and Rehabilitation of Severe Crush Injury Resulting in Lisfranc Fracture: A Case Report

**DOI:** 10.7759/cureus.54473

**Published:** 2024-02-19

**Authors:** Radha Nangliya, Sojwal P Nandanwar, Maithili Deshpande

**Affiliations:** 1 Musculoskeletal Physiotherapy, Ravi Nair Physiotherapy College, Datta Meghe Institute of Higher Education and Research, Wardha, IND

**Keywords:** physiotherapy, rehabilitation, case report, pain management, outcome evaluation, acute crush injury, lisfranc fracture

## Abstract

A 58-year-old male patient was seriously injured in his left foot as a result of a passenger car accident, resulting in a Lisfranc fracture and complications on his left side. The initial injury resulted in a 20-cm laceration, severe pain, and severe swelling. After primary care at the government hospital, the patient was referred to Acharya Vinoba Bhave Rural Hospital (AVBRH) for further treatment. Clinical examination revealed infection, Lisfranc ligament rupture, bone exposure, restriction of movement, and muscle strength. His fracture was managed with Kirschner wire(K-wire) fixation surgically. A holistic physical management plan includes immobilization and a comprehensive medical program to reduce edema, muscle atrophy, and joint stiffness. Post physiotherapy showed significant improvements in joint function, muscle strength, and functional scores after rehabilitation. Outcome measures such as the Lower Extremity Functional Scale, Olerud-Molander Ankle Score, and Patient-Reported Outcomes Measurement Information System-29 are related to physical recovery, stress reduction, and healing as a whole-life treatment. These data highlight the importance of a collaborative, multidisciplinary approach in the effective management of Lisfranc fracture injuries and confirm the advantages of timely intervention and physical therapy for the benefit of these patients.

## Introduction

Crush injuries can damage bone and soft tissue, making healing more difficult [1]. A minor injury can be treated with just ice and compression, but if swelling, pain, or bleeding is excessive, be sure to see your doctor for treatment. Treatment options include casting, splinting, physical therapy, medication, and surgery [2].

A Lisfranc injury is also known as a foot injury that occurs when one or more metatarsals are separated from the tarsal joint [3]. It is a tarsometatarsal fracture dislocation characterized by traumatic disruption between the articulation of the medial cuneiform and the base of the second metatarsal. This is a frequent foot condition, and the degree of pain varies. Injury to the foot can occur as a result of a fall risk, fracture, or direct injury to the foot [4,5]. Symptoms of a Lisfranc injury include swelling, discomfort, and heaviness in the foot. Low-energy trauma is the most common mechanism of injury, with an incidence of unstable injury of 6/100,000 person-years. A Lisfranc injury occurs in 20% of athlete patients, especially in patients who self-harm and have multiple injuries [6]. Missed and delayed diagnoses are associated with long-term disability [7].

Kirschner wire (K-wire) reduction and internal fixation are very effective and can prevent postoperative pain and improve the patient's quality of life [8]. Treatment for Lisfranc includes immobilization with a cast or boot, surgery, and physical therapy. Physical therapy can help strengthen other joints, reduce stiffness, and speed up skin tissue recovery. Physical therapy goals may include stretching and strengthening, electrical stimulation, exercise, massage, acupuncture, waxing, and taping [9]. The quantum of time it takes to recover from a Lisfranc injury depends on the rigorousness of the injury and the treatment used, but full recovery may take months to periods [10].

## Case presentation

A 58-year-old male patient was seriously injured after being hit by a returning bus on the night of September 27, 2023, and went to Acharya Vinoba Bhave Rural Hospital (AVBRH) with his left foot bandaged. He complained of severe pain, swelling, and tenderness in the middle of the foot, as well as a 20-cm incision. After initial treatment at the public hospital, he was referred to AVBRH for further treatment. Trauma history was present, with avulsion of the left ankle and fracture involving all of the cuneiform and cuboid bones.

Clinical findings

After getting consent from the patient, he was examined in a well-lit room. Vital signs were stable, with a heart rate of 90 beats per minute, a breathing rate of 20 breaths per minute, a blood pressure of 110/70 mmHg, and an afebrile temperature. General examination revealed normal higher function, decubitus, and nutritional status. The examination of the left foot showed a contaminated 20-cm lacerated wound, swelling over the ankle and distal one-third of the leg, tenderness on the dorsum, and exposed bone and tissue. Palpation revealed an elevated local temperature, tenderness over the plantar and dorsum, bony crepitus, and palpable dorsalis pedis and posterior tibial arteries. Dressing was done under sterile conditions with Betadine H_2_O_2_ and normal saline. Pain on the Numerical Pain Rating Scale was 8/10. Joint movement is shown in Table 1, and manual muscle testing is shown in Table 2. Superficial reflexes like plantar response were normal, abdominal response was intact, and deep reflexes were intact. The patient was having difficulty in daily activities like standing, walking, and toileting.

**Table 1:** Postoperative assessment findings of range of motion NA: not assessable

Joint movement	Right side	Left side
Hip flexion	0°-110°	0°-105°
Hip extension	0°-30°	0°-20°
Hip abduction	0°-25°	0°-30°
Hip adduction	0°-20°	0°-20°
Hip external rotation	0°-25°	0°-30°
Hip internal rotation	0°-25°	0°-30°
Knee flexion	0°-110°	0°-20°
Knee extension	110°-0°	20°-0°
Ankle dorsiflexion	0°-10°	NA
Ankle plantarflexion	0°-40°	NA

**Table 2:** Postoperative assessment findings of manual muscle testing by the MRC grading MRC: Medical Research Council

Manual muscle testing	Right side	Left side
Hip flexors	3/5	3/5
Hip extensors	3/5	3/5
Hip abductors	3/5	3/5
Hip adductors	3/5	3/5
Knee flexors	3/5	2/5
Knee extensors	3/5	2/5
Ankle dorsiflexors	3/5	-
Ankle plantarflexors	3/5	-

Diagnostic findings

**Figure 1:**
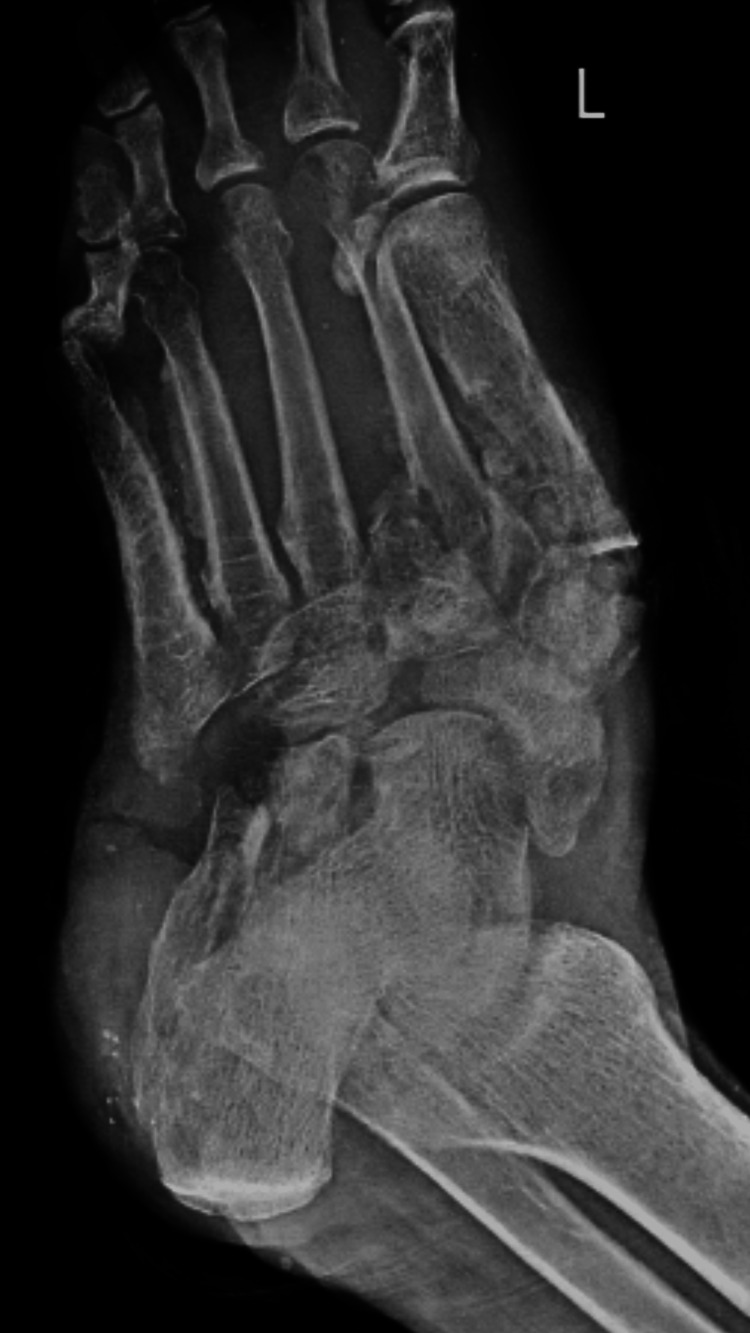
Preoperative X-ray of the ankle and toes X-ray of the ankle and toes shows a Lisfranc fracture of the left foot and all cuneiform and cuboid fractures of the left foot

**Figure 2:**
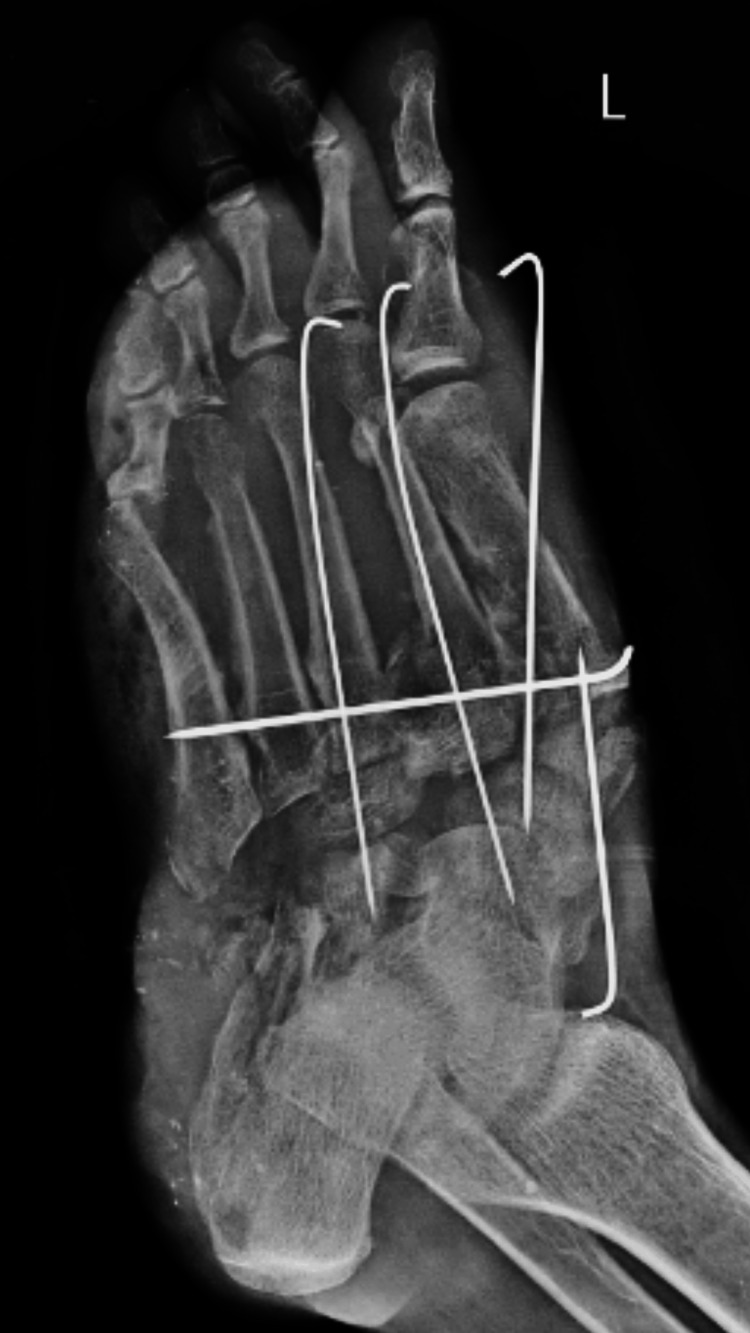
Postoperative X-ray of the ankle and toes X-ray shows the K-wire fixation of the cuneiform and cuboid fracture and horizontal K-wire fixation for the stability of the left ankle K-wire: Kirschner wire

The radiological finding of the ankle and toes shows a Lisfranc fracture with all cuneiform and cuboid fractures of the left foot (Figure 1). The patient was diagnosed with crush injury of the left foot, Lisfranc fracture, all cuneiform and cuboid fractures of the left foot, and heel pad avulsion of the left foot. The patient underwent wound debridement and K-wire fixation (Figure 2).

Physiotherapy management and pharmacological management

**Figure 3:**
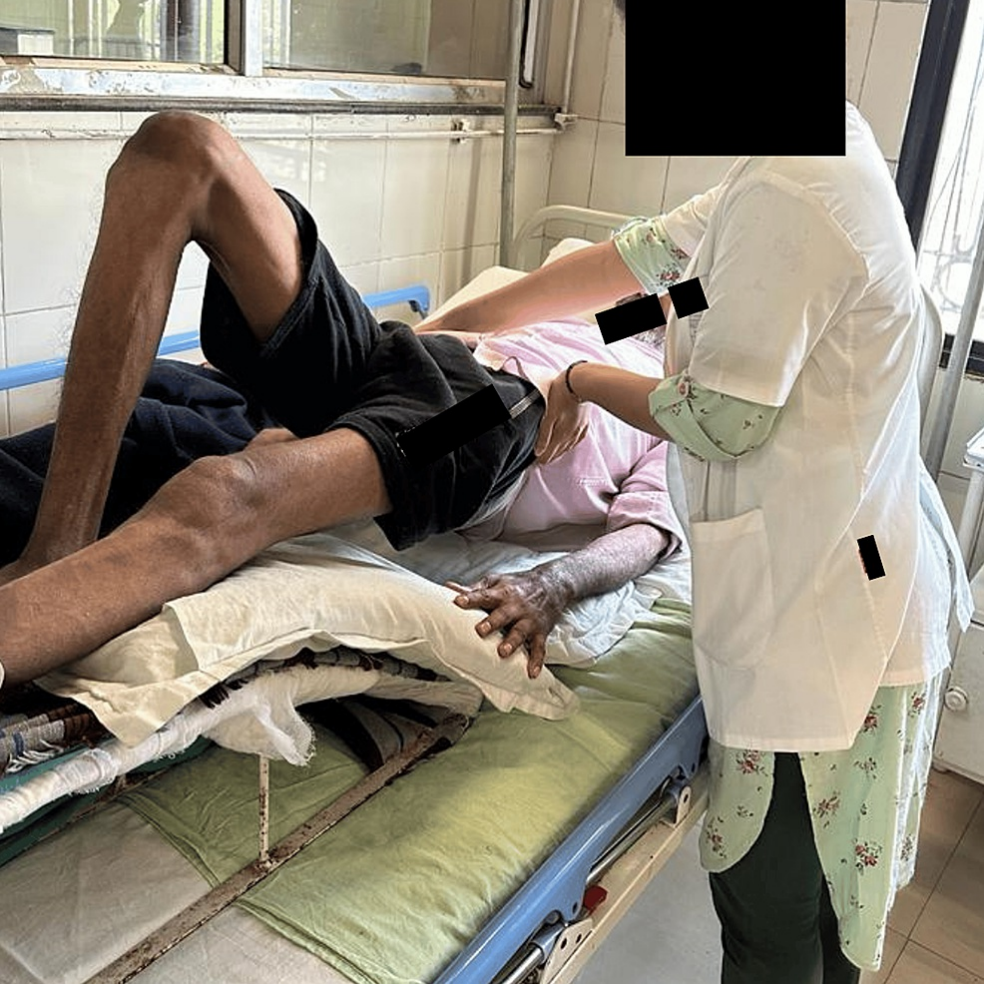
Pelvic bridging Pelvic bridging exercises are performed to improve the strength of the extensors of the lower back and hip

**Figure 4:**
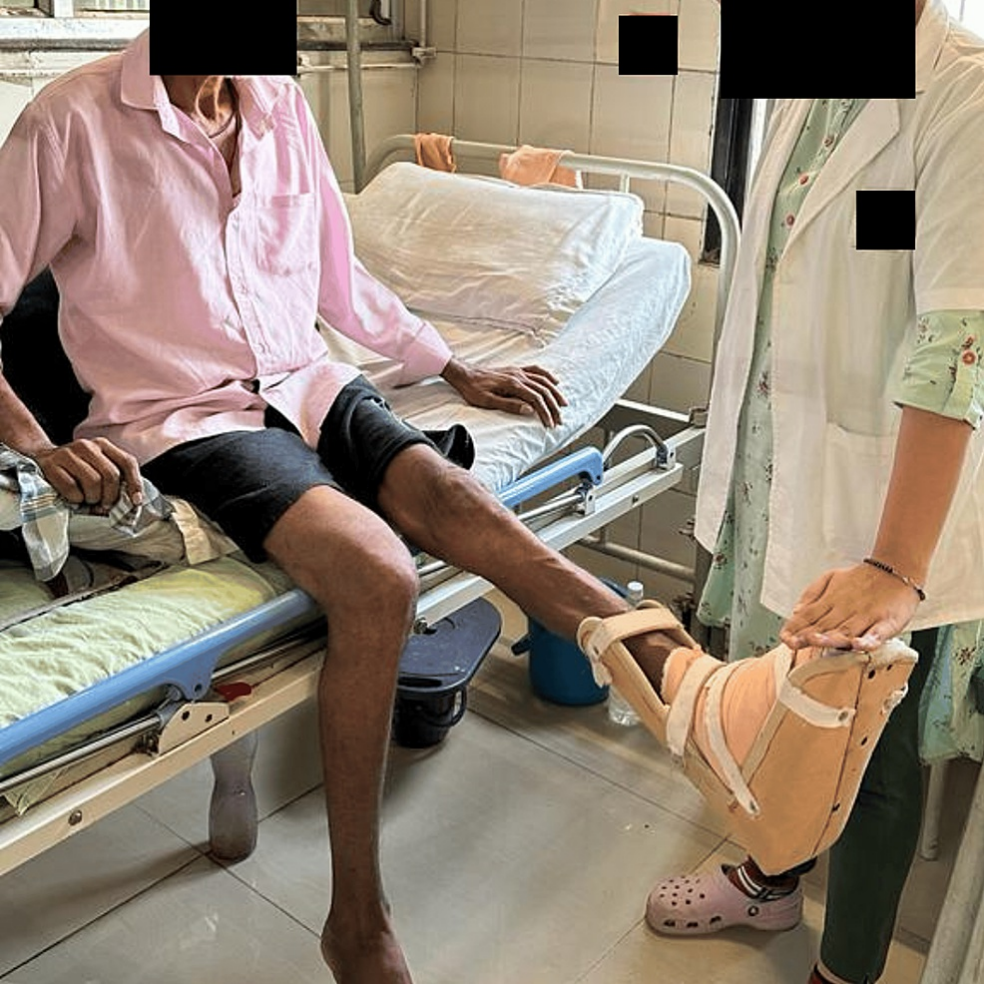
Dynamic quads of the left side Dynamic strengthening exercises are performed to strengthen the quadriceps muscle

The most popular method of treatment is to use implants of external pins or internal screws to secure the fractured and dislocated bones. Both surgical and conservative treatments start soon after immobilization, with pharmacological treatment in Table 3 and physiotherapy intervention depicted in Table 4. Interventions include reducing edema, strengthening to treat atrophy resulting from immobilization, exercises for inflexibility, and gait, and the production of bottom orthoses to support the tarsometatarsal articulations. Exercises are mentioned in Figure 3 and Figure 4.

**Table 3:** Pharmacological management Tab.: tablet; mg: milligram

Medication	Dosage
Tab. ibuprofen 400 mg	Twice daily
Tab. vitamin C 500 mg	Twice daily
Tab. trypsin+chymotrypsin I	Thrice daily

**Table 4:** Physiotherapy protocol ROM: range of motion; AROM: active range of motion; PROM: passive range of motion; FWB: full weight bearing; kg: kilogram

Days and weeks	Goals	Intervention	Dosage and progression
Phase I: weeks 1-2	Rest and recovery from surgery. Reduce swelling and discomfort. Instructions for the safe and proper use of crutches/rolling crutches to improve daily living activities.	Precautions should be taken, such as not bearing weight and standing on one leg when showering, but not walking. Family and patient education on surgical procedure, anatomy, and healing time. AROM in the hip. AROM in the knee splint.	10 reps × 2 sets and then increase to 10 reps × 4 sets
Phase II: weeks 3-6	Maintain hip and knee ROM. Strengthen your core, hips, and knees. Rolling crutches or crutches are used safely at the protected fusion site.	Cam boot or fibreglass casting safeguards the graft and enables you to bear weight on your toes when standing to carry out daily tasks. Elevate to reduce swelling. Abdominal recruitment exercises include ball reach and bridging. Strengthening exercises include straight leg raises with holds, AROM in the hip, and AROM in the knee. At 6-8 weeks, depending on the surgeon's assessment, AROM with ankle dorsiflexion/plantarflexion and inversion/eversion may be suggested. Stretching of the gluteus maximus, piriformis, rectus femoris, and hamstrings.	10 reps × 2 sets
Phase III: weeks 6-10	Walker boots with FWB boost your knee, hip, and core strength.	Elevation to reduce edema on a stationary cycle proceeds with strengthening your core, hips, and knees using manual resistance and a 0.5-kg weight cuff. Progressive FWB during walker boot.	10 reps × 4 sets
Phase IV: weeks 11-12	FWB without booting.	Apply massage to reduce swelling. AROM-like inversion/eversion of the ankle dorsiflexors and plantarflexors. As needed to begin gait retraining, the muscles of investors and evertors should be stimulated. Advance workouts to standing leg press depending on the fused joint; wean from the walker boot (may start sooner depending on the surgeon's assessment).	10 reps × 2 sets and then increase to 10 reps × 4 sets
Phase V: weeks 13-15	The ROM in full for non-fused joints is almost at maximum power, the ideal pattern of gait.	AROM and PROM in non-fused joints and the ankle. Stretching of the gluteus, piriformis, hamstrings, calf, and rectus femoris. Manual mobilization of the ankle, foot, and toes' limited non-fused joints. Using fusion to retrain gait to optimal mechanics, ankle strength training using Theraband, non-weight bearing dorsiflexors and inversion/eversion, weight bearing inversion/eversion, and toe raises. Proprioceptive education advancement like a single-leg stance on a wobble board, single-leg stance on a level surface, and Sissel or double-leg stance could possibly receive an ankle brace.	10 reps × 2 sets and then increase up to 10 reps × 4 sets
Phase VI: weeks 16	Complete strength and complete functionality for work.	Strength training carries on with the activities from week 15. Week 15's proprioceptive training was previously mentioned. Proceed retraining one's gait with orthotics or, if necessary, modifying one's shoes.	10 reps × 4 sets

Outcome measures and follow-up

Outcome measures are mentioned in Tables 5-7. During discharge, the home program was taught to the patient, and he came for a follow-up to check his progress.

**Table 5:** Outcome measures

Scales	Pre rehabilitation (day 3)		Post rehabilitation (weeks 8-12)	
Lower Extremity Functional Scale	26/80		58/80	
Patient-Reported Outcomes Measurement Information System-29	Physical function	8/20	Physical function	16/20
	Anxiety	12/20	Anxiety	0/20
	Depression	12/20	Depression	0/20
	Fatigue	16/20	Fatigue	3/20
	Sleep disturbance	4/20	Sleep disturbance	0/20
	Ability to perform the activity	16/20	Ability to perform the activity	5/20
	Pain interference	12/20	Pain interference	3/20
	Pain intensity	8/10	Pain intensity	1/10
Olerud-Molander Ankle Score	10/100		75/100	

**Table 6:** Post-rehabilitation findings of manual muscle testing Pre-rehabilitation assessment on day 7 and post-rehabilitation assessment on week 3

Manual muscle testing	Pre rehabilitation		Post rehabilitation	
Muscles	Right	Left	Right	Left
Hip flexors	3/5	3/5	5/5	5/5
Hip extensors	3/5	3/5	5/5	5/5
Hip abductors	3/5	3/5	5/5	5/5
Hip adductors	3/5	3/5	5/5	5/5
Knee flexors	3/5	2/5	5/5	4/5
Knee extensors	3/5	2/5	5/5	4/5
Ankle dorsiflexors	3/5	2/5	5/5	4/5
Ankle plantar flexors	3/5	2/5	5/5	4/5

**Table 7:** Post-rehabilitation findings of range of motion Pre-rehabilitation assessment on day 7 and post-rehabilitation assessment on week 5

Range of motion	Pre rehabilitation		Post rehabilitation	
Joints	Right	Left	Right	Left
Hip flexion	0°-110°	0°-105°	0°-120°	0°-115°
Hip extension	0°-30°	0°-20°	0°-30°	0°-30°
Hip abduction	0°-25°	0°-30°	0°-30°	0°-30°
Hip adduction	0°-20°	0°-20°	0°-25°	0°-25°
Hip external rotation	0°-25°	0°-30°	0°-45°	0°-40°
Hip internal rotation	0°-25°	0°-30°	0°-45°	0°-45°
Knee flexion	0°-110°	0°-20°	0°-130°	0°-100°
Knee extension	110°-0°	20°-0°	130°-0°	100°-0°
Ankle dorsiflexion	0°-10°	0°-5°	0°-20°	0°-15°
Ankle plantarflexion	0°-40°	0°-10°	0°-50°	0°-40°

## Discussion

The presented case involves a 58-year-old male who suffered a crush injury to his left foot, leading to a Lisfranc fracture and additional complications on the left foot. The management and rehabilitation of such injuries involve a multidisciplinary approach, including medical, surgical, and physiotherapeutic interventions. The physiotherapy management plan incorporates a combination of immobilization, surgical intervention, and rehabilitation exercises [11]. In cases of mild sprains without diastasis, immobilization with a cast or boot is recommended. However, in this severe Lisfranc fracture involving all cuneiform and cuboid bones, surgical fixation with internal screws or external pins is the suggested treatment [12]. Physiotherapy interventions play a crucial role in the postoperative and conservative phases of treatment [13]. Early interventions focus on edema reduction, addressing muscle atrophy, and maintaining joint flexibility [14]. The presented physiotherapy program includes a range of exercises, electrotherapy techniques, mobilizations, massage, acupuncture, wax therapy, and taping [15]. They employed a sonic or balance pad for proprioception training and kept their balance while bending the knee of one leg 90 degrees on their own [16]. Strength training and balance training have been used for better patient outcomes in individuals with a history of ankle sprains [17]. These interventions aim to keep other joints strong, reduce stiffness, and accelerate tissue healing. The presented outcome measures include the Lower Extremity Functional Scale [18] and the Patient-Reported Outcomes Measurement Information System-29 [19]. The Olerud-Molander Ankle Score is used to evaluate the functional outcome based on the postoperative treatment of ankle fractures [20]. The post-rehabilitation scores demonstrate a substantial improvement in almost all categories, indicating a positive response to the integrated treatment approach. The case provides pre-rehabilitation and post-rehabilitation outcomes, showcasing advances in joint movement, manual muscle testing, and functional scales. Strength, functional ability, and flexibility in motion significantly improve after rehabilitation, indicating the effectiveness of the physiotherapeutic interventions.

## Conclusions

The comprehensive management of crush injuries involving Lisfranc fractures requires collaboration between medical professionals and physiotherapists. The presented case highlights the importance of timely intervention, surgical fixation, and structured physiotherapy programs in achieving optimal functional outcomes and enhancing the quality of life for the patient. The success of rehabilitation is evident in the improved joint movements, muscle strength, and outcome measure which shows positive results after giving physiotherapy.
